# Characterization of Low-Molecular-Weight Dissolved Organic Matter Using Optional Dialysis and Orbitrap Mass Spectrometry

**DOI:** 10.3390/molecules29143370

**Published:** 2024-07-18

**Authors:** Qiuxing Li, Runyu Zhang, Guopei Huang, Haijun Yuan, Liying Wang, Shuxia Xu

**Affiliations:** 1 Institute of Geochemistry, Chinese Academy of Sciences, Guiyang 550081, China; liqiuxing1021@163.com (Q.L.); huangguopei@mail.gyig.ac.cn (G.H.); yuanhaijun@mail.gyig.ac.cn (H.Y.); wangliying@vip.gyig.ac.cn (L.W.); 2 College of Earth Science, Chengdu University of Technology, Chengdu 610059, China; xushux@cdut.edu.cn; 3 University of Chinese Academy of Sciences, Beijing 100049, China

**Keywords:** dissolved organic matter, low-molecular-weight fraction, membrane dialysis, Orbitrap MS, van Krevelen diagram

## Abstract

Low-molecular-weight (LMW, <1000 Da) dissolved organic matter (DOM) plays a significant role in metal/organic pollutant complexation, as well as photochemical/microbiological processes in freshwater ecosystems. The micro size and high reactivity of LMW-DOM hinder its precise characterization. In this study, Suwannee River fulvic acid (SRFA), a commonly used reference material for aquatic DOM, was applied to examine the optical features and molecular composition of LMW-DOM by combining membrane separation, ultraviolet–visible absorption and Orbitrap mass spectrometry (MS) characterization. The 100–500 Da molecular weight cut-off (MWCO) membrane had a better performance in regard to separating the tested LMW-DOM relative to the 500–1000 Da MWCO membrane. The ultraviolet–visible absorbance decreased dramatically for the retentates, whereas it increased for the dialysates. Specifically, carbohydrates, lipids and peptides exhibited high selectivity to the 100–500 Da MWCO membrane in early dialysis. Lignins, tannins and condensed aromatic molecules displayed high permeability to the 500–1000 Da MWCO membrane in late dialysis. Overall, the retentates were dominated by aromatic rings and phenolic hydroxyls with high O/C_wa_ (weighted average of O/C) and low H/C_wa_. Conversely, such dialysates had numerous aliphatic chains with high H/C_wa_ and low O/C_wa_ compared to SRFA. In particular, LMW-DOM below 200 Da was identified by Orbitrap MS. This work provides an operational program for identifying LMW-DOM based on the SRFA standard and MS analysis.

## 1. Introduction

Dissolved organic matter (DOM) is a class of heterogeneous organic mixtures with different molecular weights and plays a crucial role in the biotic and abiotic processes of aquatic environments [1,2,3]. For instance, DOM (e.g., C2, C3 and C4) has explained over 11.8% of the variation in phytoplankton communities under low-flow velocity conditions, which even exceeds the influence of major nutrients, like total nitrogen and total phosphorus [4]. In recent decades, DOM has concerned ecologists, since its high concentration substantially aggravates the “color” of natural waters [5,6]. Owing to anthropogenic activities and terrigenous inputs, large amounts of DOM flowing into aquatic environments have led to the wild proliferation of phytoplankton and the overgrowth of aquatic plants [1,7], which pose a considerable threat to biodiversity [8]. Indeed, DOM contains not only organic carbon but also organic nitrogen, sulfur and phosphorus. After microbial mineralization, DOM provides sufficient nutrients, including carbon, nitrogen and phosphorus sources, to promote the growth of aquatic organisms like bacterioplankton and phytoplankton [8,9]. As such, DOM serves as an essential source of nutrients and energy for organisms and regulates ecological functions and nutrient status [7,10]. In this context, it is beneficial to unveil the molecular size, structure and molecular composition of DOM in various aquatic environments [11].

A knowledge of DOM based on the different sizes or molecular weight fractions can deepen our understanding of bio-organic geochemistry [12]. According to its molecular size, DOM can often be partitioned into high-molecular-weight DOM (HMW-DOM; >1000 Da) and low-molecular-weight DOM (LMW-DOM; <1000 Da) [13]. The former has a higher biological stability than the latter, implying that LMW-DOM is more susceptible to microbial degradation than HMW-DOM [14]. Compared to HMW-DOM, LMW-DOM also exhibits a greater potential to improve the bioavailability of metal elements, such as cadmium and hydrargyrum, to marine bivalves [15]. More interestingly, in algae and aquatic plants, LMW-DOM, rather than HMW-DOM, exhibits a high potential for the photochemical formation of reactive oxygen species [16]. Traditional theory has confirmed that DOM is dominated by relatively large molecules through gel filtration and ultrafiltration [11]. By contrast, there is overwhelming evidence that DOM contains excess supramolecular assemblies of small molecular compounds that enhance structural stability through their hydrophobic effect and hydrogen bonds [17,18]. Although HMW-DOM acts as a recalcitrant molecule, it is also prone to releasing LMW compounds through photodegradation [19]. Accordingly, LMW-DOM contains numerous complex compounds with different molecular sizes and chemical structures, which comprise substantial proportions of the DOM in freshwater ecosystems [3]. During the chlorination of drinking water, LMW-DOM not only affects the water color and odor but also forms various harmful disinfection by-products [20]. Thus, there is an urgent need to explore the molecular composition of LMW-DOM to enhance our understanding of its toxicity and bioavailability, and to subsequently develop strategies to protect aquatic environments.

Currently, the molecular weight distribution of DOM is mostly characterized using viscosity-based methods, ultrafiltration, dialysis, low-angle X-ray scattering, and high-performance size-exclusion chromatography or counter-current chromatography [11,21,22]. In fact, combining reverse osmosis, cross-flow ultrafiltration and size-exclusion chromatography with conventional spectroscopy techniques, like ultraviolet–visible absorption spectroscopy (UV-Vis) and excitation–emission matrix fluorescence (EEM), can effectively reveal the chromophoric and fluorescent properties of LMW-DOM [23]. Furthermore, Fourier-transform ion cyclotron resonance (FT-ICR) and Orbitrap mass spectrometry (MS) hold great potential for characterizing the molecular weight of DOM in aquatic environments due to their high resolution and mass accuracy. Compared to FT-ICR, Orbitrap MS is more suitable for analyzing the composition of LMW-DOM. A majority of intense masses in the 100–290 mass-to-charge ratios (*m*/*z*) range were detected by Orbitrap MS for SRFA with an aliphatic characteristic [11]. Subsequent studies have shown that the high resolving power of Orbitrap MS can extend masses down to 50 Da for the characterization of the LMW-DOM in SRFA or a lake (Plåten, Sweden) [24,25]. Furthermore, Vaughn Mangal et al. successfully characterized aquatic DOM samples with low N and S proportions and LMW-DOM in SRFA, Pony Lake fulvic acid (PLFA) and phytoplankton (*Scenedesmus obliquus*, *Euglena mutabilis* and *Euglena gracilis*) [26]. Admittedly, the emergence of high-resolution MS technologies has largely filled the knowledge gap regarding DOM and has shed light on its importance to aquatic ecosystems. However, it is still imperative for us to further examine the chemical composition and molecular structure of LMW-DOM in aquatic environments, particularly that with a molecular weight fraction below 200 Da.

In view of the diversity of DOM sources and the heterogeneity of their molecular compositions, a number of reference standards are commonly used for DOM-related studies in different environments. Among them, SRFA is one frequently used surrogate with which to represent aquatic DOM [27,28]. Extensive research has been conducted on the characterization of SRFA by capillary electrophoresis [29], UV−Vis [30], flow field–flow fractionation and EEM [31], and ^13^C Nuclear Magnetic Resonance and Electron Paramagnetic Spectroscopic [32]. However, revealing the molecular composition of LMW-DOM based on SRFA molecular weight fractionation and MS analysis is still lacking [11]. For this reason, this study aimed to investigate the molecular composition of LMW-DOM using membrane dialysis and Orbitrap MS analysis. Here, we selected two molecular weight cut-off (MWCO) membranes (i.e., 100–500 Da and 500–100 Da) to perform dialysis with SRFA as a well-characterized reference standard. The initial SRFA solution, dialysate and retentate were then analyzed using UV-Vis absorbance (BlueStar A, LabTech, Sorisole, Italy) and Orbitrap MS analysis. Furthermore, the effectiveness of Orbitrap MS in characterizing the LMW-DOM components at below 200 *m*/*z* range was verified. This work provides an available scheme for assessing the dynamic behavior and molecular composition of LMW-DOM in aquatic environments by coupling membrane dialysis with high-resolution MS analysis.

## 2. Results

### 2.1. DOC Concentrations and UV−Vis Characteristics of the Retentate or Dialysate

**Figure 1:**
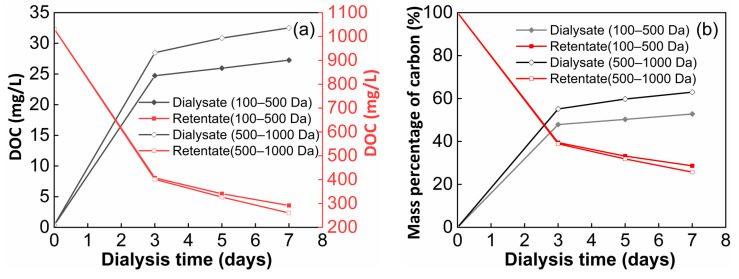
Variations in dissolved organic carbon concentration’s (**a**,**b**) mass percentage in the SRFA retentate and dialysate using two kinds of cut-off membranes.

The changes in dissolved organic carbon (DOC) concentration throughout the dialysis experiment are shown in Figure 1. The DOC concentration significantly decreased from 1032 (initial SRFA solution) to 405 mg·L^−1^ in the retentate for two kinds of cut-off membranes on the third day but increased to 25 and 28 mg/L in the dialysate for the 100–500 Da and 500–1000 Da MWCO membranes, respectively (Figure 1a). As the dialysis proceeded, the DOC concentration in the retentate was reduced to 291 and 260 mg/L on the seventh day but was raised slightly to 27 and 33 mg/L in the dialysate for the 100–500 Da and 500–1000 Da MWCO membranes, respectively. Based on the calculation of varying DOC concentrations over the experimental periods, the apparent retention rate declined from 39% to 29% and 26% for the two kinds of dialysis membranes from the third to seventh days (Figure 1b). In other words, a significant fraction (53% and 63% based on the DOC mass) of SRFA eventually passed through the two kinds of cut-off membranes, regardless of the changes in volume for the retention solutions.

**Figure 2:**
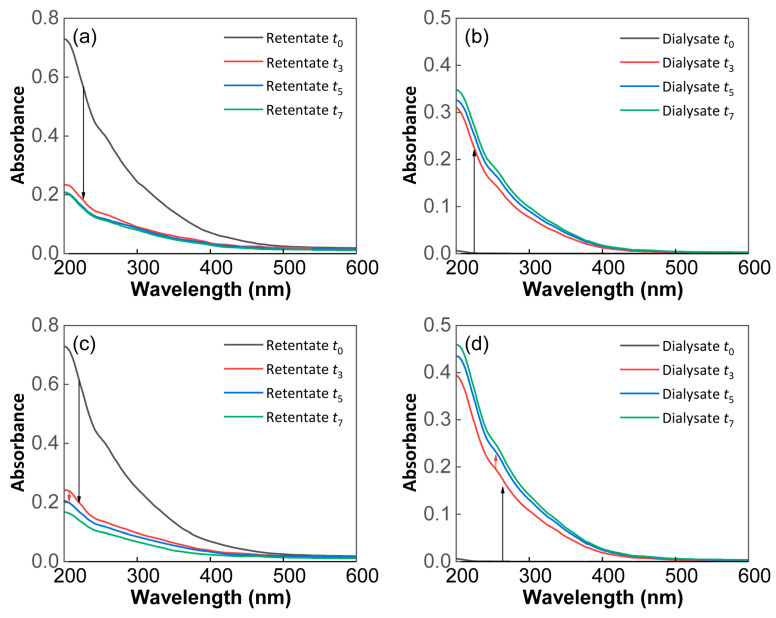
UV−Vis absorption spectra of the SRFA retentate and dialysate. (**a**) The retentate and (**b**) dialysate of the 100–500 Da MWCO membrane. (**c**) The retentate and (**d**) dialysate of the 500–1000 Da MWCO membrane. *t*_0_, *t*_3_, *t*_5_ and *t*_7_ stand for the samples from the zeroth, third, fifth and seventh days of the dialysis experiment. The retentate (*t*_0_, *t*_3_, *t*_5_ and *t*_7_) measurements were obtained by diluting the original solution with Milli-Q water by 1/300×, 1/240×, 1/200× and 1/160×, while the dialysate (*t*_3_, *t*_5_ and *t*_7_) measurements were obtained by diluting the original solution with Milli-Q water by 1/2×, but their absorbances were calculated back to those of the undiluted samples.

The UV−Vis absorption spectra of the SRFA retentate and dialysate showed significantly different trends (Figure 2). Overall, the UV−Vis absorbance decreased dramatically for the SRFA retentate but increased for the dialysate during the dialysis. The initial SRFA solution had obvious UV–Vis absorption peaks in the 200–500 nm wavelength range. With increasing dialysis time, the UV−Vis absorbance at 254 and 280 nm decreased significantly for the SRFA retentate, starting from the third day (Figure 2a,c), but increased sharply for the dialysate (Figure 2b,d). Although the UV−Vis absorption spectra of the SRFA retentate and dialysate showed similar trends between the two kinds of cut-off membranes, the amplitude of the fluctuations was greater for the 500–1000 Da MWCO membrane.

In addition, the characteristic parameters of the UV–Vis absorption spectra are summarized in Supplementary Table S1. Moreover, *a*_254_ and *a*_280_ rapidly decreased in the SRFA retentate of the two kinds of dialysis membranes after the dialysis but increased gradually for the dialysate, with a larger range of variation for the 500–1000 Da MWCO membrane than the 100–500 Da MWCO membrane. SUVA_254_ and SUVA_280_ in the SRFA retentate declined in the first three days and then later increased slightly, but for the SRFA dialysate, they increased continuously throughout the entire dialysis duration. Moreover, *E*2/*E*3, *E*3/*E*4 and *E*4/*E*6 ratios showed slight decreasing trends for the SRFA retentate but opposite trends for the SRFA dialysate, especially *E*3/*E*4.

### 2.2. MS and Van Krevelen Plots of the SRFA Retentate and Dialysate

A high degree of similarity was found between the two dialysis membranes for DOM fractionation. As an example, the full MS spectra of the SRFA retentate at different dialysis intervals for the 100–500 Da MWCO membrane are shown in Supplementary Figure S1. A higher number of MS peaks was detected in the retentate on the third day (Supplementary Figure S1a). As the experiment progressed, the number of detected MS peaks gradually decreased (Supplementary Figure S1b,c). Moreover, the intensities of the MS peaks at *m*/*z* 97.0046, 129.9765, 146.9639, 242.9442, 316.9482 and 378.9345 were significantly weakened with the increasing dialysis time. The full MS spectra of the SRFA dialysate on the fifth day from the two kinds of cut-off membranes are exhibited in Supplementary Figure S2a,b. The *m*/*z* of both membranes was concentrated in the 200–500 Da range for the dialysates and resembled that of the SRFA retentates (Supplementary Figure S1a–c).

In order to distinguish the differences between the dialysates of the two kinds of membranes (Supplementary Figure S2a,b), we zoomed in on the LMW interval of the 70–200 Da membrane. As shown in Supplementary Figure S2c,d, the MS peak intensities of the dialysates differed for the 100–500 Da and 500–1000 Da MWCO membranes at *m*/*z* 184.9842, 172.9723, 154.9613, 146.9602, 129.9854, 102.9486, 97.0136, etc. Generally, the LMW compounds from the dialysate of the 100–500 Da membrane had stronger peaks and more molecular compositions than those of the 500–1000 Da membrane.

**Figure 3:**
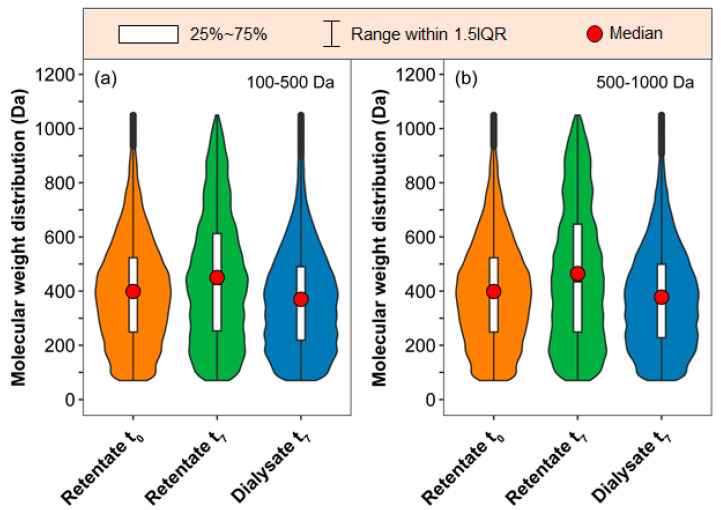
Molecular weight distribution and abundance of DOM compounds detected in the initial solution, and final retentate and dialysate from the (**a**) 100–500 Da and (**b**) 500–1000 Da MWCO membranes. The fat and thin fusiform shapes represent more and less content, respectively. The dark vertical line in the center symmetrically divides the figure in order to conveniently observe the fat and thin portions of the figure. The ends of the white bars and the red circles represent the quartiles of the DOM’s molecular weight distribution.

Furthermore, a few differences between the two kinds of dialysis membranes were visible in the mass–charge ratio distribution (Figure 3). As expected, the SRFA retentate of the 100–500 Da membrane had less HMW compounds (>500 Da, particularly over 700 Da) than that of the 500–1000 Da membrane after 7 days of dialysis. Additionally, as marked by the red dots, the mass distributions of the SRFA retentates from the 100–500 Da and 500–1000 Da MWCO membranes were mostly concentrated at 428 Da and 437 Da, respectively. Interestingly, more LMW DOM compounds below 500 Da were identified in the dialysate of the 100–500 Da MWCO membrane on the seventh day (Supplementary Table S2).

The van Krevelen (VK) diagram shows the identified DOMs in the original SRFA solution, with the colors representing the normalized peak strengths, and also serves as a heat map (Supplementary Figure S3). The SRFA mainly consisted of lipids, proteins (including peptides), carbohydrates, unsaturated hydrocarbons, lignins, tannins and condensed aromatic molecules. Among them, lignins and tannins had the highest normalized strengths, followed by some condensed aromatic molecules, proteins and carbohydrates, while the compounds with the lowest normalized strength were lipids.

The VK plots with full *m*/*z* 70–800 provide the temporal changes in DOM distributions between the two kinds of cut-off membranes (Supplementary Figure S4). The DOM compounds remaining in the retentate on the third day were mainly lignins, tannins and condensed aromatic molecules. In contrast, in the 100–500 Da MWCO membrane, less proteins/peptides (6) and carbohydrates (2) remained than in the 500–1000 Da MWCO membrane (11 and 5, respectively). Hereafter, no significant differences were found for the H/C and O/C values in the retentate until the seventh day, which was consistent with the results of the original MS spectra (Supplementary Figure S1). On the seventh day, the 500–1000 Da MWCO membrane had less lignins (375), tannins (43) and condensed aromatic molecules (36) than the 100–500 Da MWCO membrane (388, 61 and 69, respectively).

A rich variety of DOMs were concurrently detected in the dialysate of the two kinds of membranes on the third day, similar to those in the retentate (Supplementary Figure S5a,b). The dialysate from the 100–500 Da MWCO membrane contained more proteins/peptides (71) and carbohydrates (18) than the 500–1000 Da MWCO membrane (55 and 7, respectively). Stable H/C and O/C distributions indicated that the dialysate DOM composition changed slightly after the third day (Supplementary Figure S5c–f). Finally, the dialysate from the 500–1000 Da MWCO membrane contained more lignins (1521) and tannins (280) than the 100–500 Da MWCO membrane (1326 and 234, respectively).

**Figure 4:**
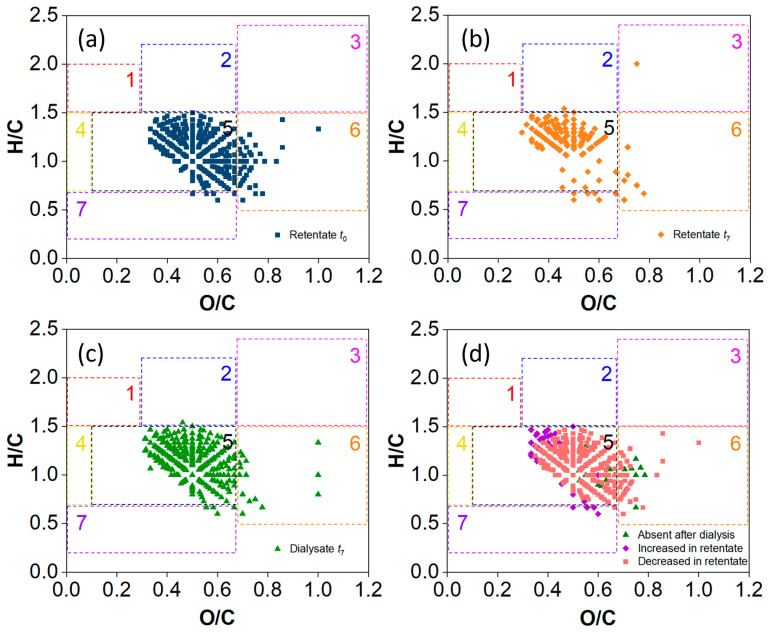
VK plots of the identified DOM compounds with *m*/*z* 70–800 in the SRFA solution for the 100–500 Da MWCO membrane. (**a**) The initial SRFA solution. (**b**,**c**) The retentate and dialysate, respectively, on the seventh day. (**d**) Changes in the DOM compounds in the SRFA solution after dialysis. Only compounds with normalized magnitudes higher than 0.001 in the mass spectra are shown, representing between 13% and 23% of the corresponding identified peaks. The numbers in the figures represent (1) lipids, (2) proteins (including peptides), (3) carbohydrates, (4) unsaturated hydrocarbons, (5) lignins, (6) tannins and (7) condensed aromatic molecules.

The distribution of DOM compounds in the original SRFA solution with a normalized magnitude above 0.001 was investigated for its intensity changes during the dialysis process (Figure 4a). On the seventh day, the DOM constituents showed a bias toward lower O/C and higher H/C distributions in the SRFA retentate of the 100–500 Da MWCO membrane (Figure 4b), while the tannins favored a higher O/C ratio in the dialysate (Figure 4c). In general, the abundance of most lignins and tannins decreased, while a small portion of them was removed through membrane dialysis, accompanied by an increase in the other lignins and condensed aromatic molecules with low O/C and high H/C values (Figure 4d). Similar variations in DOM composition were observed throughout the dialysis period for the 500–1000 MWCO membrane (Supplementary Figure S6).

**Figure 5:**
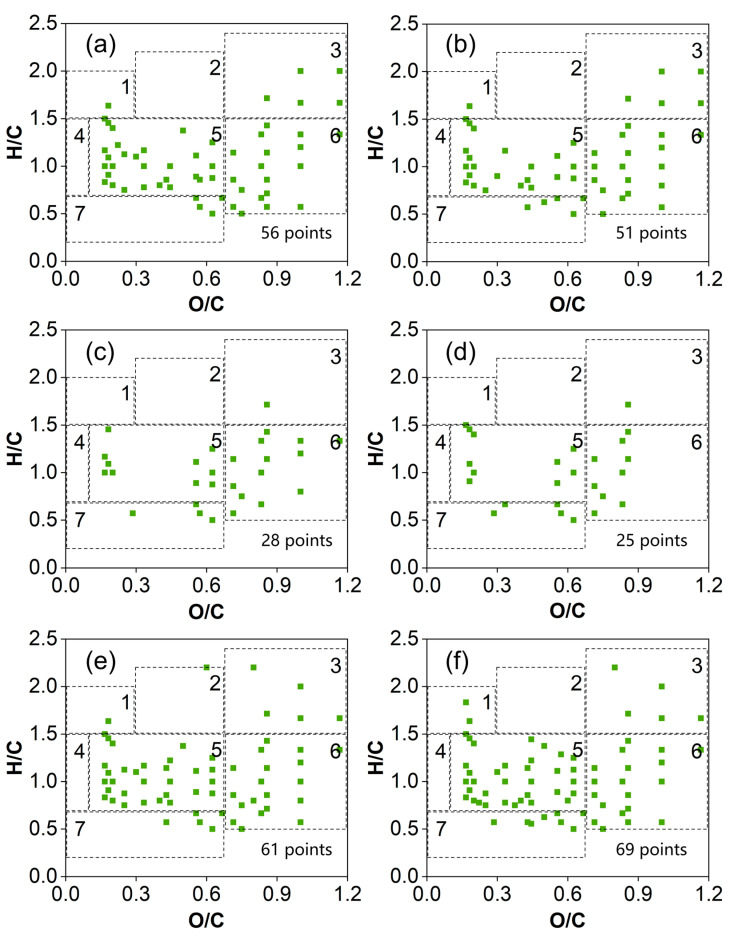
VK plots for the identified compounds with *m*/*z* below 200 in the SRFA solution. (**a**,**b**) The initial SRFA solution. (**c**,**e**) The retentate and dialysate from the 100–500 Da MWCO membrane, and (**d**,**f**) the retentate and dialysate from the 500–1000 Da MWCO membrane on the seventh day. The compounds were classified (1–7) as noted above, while the number of points is the formula numbers.

In order to track changes in the LMW DOM compounds, the identified compounds with *m*/*z* below 200 were investigated emphatically, using VK plots (Figure 5). Fifty-six and fifty-one LMW DOM compounds were identified in the original SRFA solution (Figure 5a,b). On the seventh day, half the amount of LMW DOM compounds (28 and 25) was retained in the 100–500 Da and 500–1000 Da MWCO membranes, respectively (Figure 5c,d). However, the number of LMW DOM compounds (61 and 69) significantly increased in the dialysate of the two membranes (Figure 5e,f). All of the formulas for the identified DOMs are listed in Supplementary Tables S4–S9, including those of the CHO, CHON, CHOS and CHONS compounds. The CHO formulas dominated the LMW DOM compounds, followed by the CHON formulas.

## 3. Discussion

### 3.1. Variations in the DOC Concentrations and Optical Parameters of the UV–Vis Spectra in the Retentate and Dialysate

The DOC concentrations and their relative mass percentages in the SRFA retentate and dialysate constantly changed as the dialysis experiment proceeded (Figure 1). The dialysance rate of the 500–1000 Da MWCO membrane was always higher than that of the 100–500 Da MWCO membrane, indicating that the 500–1000 Da membrane greatly facilitated the separation of humic molecules and chromophoric DOMs. Compared with a previous publication (36%) [11], our study showed a higher dialysance rate (63%) for the 500–1000 Da MWCO membrane. Correspondingly, a 43–45% DOC from freshwater DOMs has been reported to show great potential in penetrating the 500 Da cut-off membrane [33]. Therefore, our results were effective at fractionating different molecular-weight DOMs using dialysis membranes.

Moreover, a quantitative assessment of DOC mass balance could monitor the loss of DOMs during the dialysis. The total DOC mass, including the retentate and dialysate, accounted for 82% and 89% of the original SRFA for the 100–500 Da and 500–1000 Da MWCO membranes, respectively, indicating a loss of a portion of DOM constituents through the dialysis process. This was mainly attributed to changes in the volume of the retention and dialysate during the dialysis, the properties of the cut-off membrane and an alteration in the DOM organic matter itself. First, multiple instances of pipetting of the retention solution and dilution by replenishing the dialysate throughout the experiment resulted in an unavoidable underestimation of the DOC quality. Second, the type of dialysis membrane might be an important factor in determining the various DOMs. Xu et al. conducted a membrane dialysis of DOMs using three kinds of composite polyamide membranes, namely NF90 (Dow/Filmtec), NF270 (Dow/Filmtec) and HL (GE Osmonics), with corresponding MWCOs of 200, 170–300 and 150–300 Da [34]. The overall recovery of humic acid for NF90, NF270 and HL after the experiment was 67%, 89% and 83%, respectively, implying a considerable mass loss (11–33%). The different dialysis media could have influenced the carbon mass balance as well. When deionized water was used as the dialysis medium, more than 90% of the DOMs were obtained for surface water, but a slight carbon loss of about 10% was seen for wastewater due to self-adsorption onto the membrane or degradation [35]. The substrate concentrations might have also affected the carbon mass balance budget. For the dialysis in Millipore water, the carbon loss was below 5.0 ± 1.0% when the DOC concentration was less than 50 mg·L^−1^, whereas the carbon loss was up to 20.0 ± 6.0% when the DOC concentration increased [36].

As shown in the UV−Vis absorption spectra of the retentate or dialysate (Figure 2), characteristic peaks of acromion-like and protein-like substances were observed at 254 and 280 nm, which originated from the lignin and amino acids [37]. The sharp decreases in *a*_254_ and *a*_280_ in the SRFA retentate reflected the preferential removal of conjugated olefins, unsaturated carbonyl compounds, aromatics and amino acids via the dialysis process. Moreover, the aromatics dialyzed at a faster rate than the proteins, particularly for the 500–1000 Da membrane, a finding supported by the trends in SUVA_254_ and SUVA_280_ (Supplementary Table S1). Absorption ratios, defined as the ratio of absorption coefficients at two different wavelengths, are often used to unveil DOM sources and their composition characteristics in aquatic environments [38]. The *E2/E3* ratio here was inversely correlated with the molecular size of the DOMs [39]; *E*3/*E*4 and *E*4/*E*6 were both good indicators of the humification degree and aromaticity of DOMs [40,41]; and the general decreasing tendency of *E*2/*E*3, *E*3/*E*4 and *E*4/*E*6 for the SRFA retentate implied the predominance of more aromatic substances with relatively high molecular weights (Supplementary Table S1). As a result, LMW DOM molecules with more aliphatic components are more enriched in the dialysate than in the initial solution. This finding agrees with the fact that LMW DOM compounds with absorptions at low UV wavelengths often preferentially pass the cut-off membrane [11].

### 3.2. Comparison of the Mass Spectral Characteristics of the SRFA Retentate and Dialysate

A molecular-level analysis of the DOM compositions helped to elucidate their geochemical behaviors and eco-environmental function as active components of the carbon cycle [25,37,42,43,44]. In general, MS had become an indispensable and powerful tool for characterizing DOMs [42,43,44,45,46,47,48]. Humic substances are a common standard for evaluating MS methods [11,46,47], and SRFA (2S101F) from the blackwater of the Suwannee River was selected for this study. Table 1 summarizes the relevant published studies on the molecular composition of this SRFA standard using Orbitrap MS (Thermo Scientific, Waltham, MA, USA). The number of molecules identified with Orbitrap MS ranged from 2280 to 3675, with a resolution below 480,000. Specifically, the number of DOM compounds detected with Orbitrap MS (3935) in this study was slightly higher than those in other studies (1870–3675) at the same resolution, even for the same mass spectrometer model. Increasing the resolution of the mass spectrometer could result in more DOM compounds being identified [46]. However, the *m*/*z*_wa_ (355 Da) of SRFA in this work was closer to those of previously reported results (369 to 495 Da), probably because more LMW compounds were detected in our study, due to our lower scanning range (70–1050 Da), than in previous studies (150–1000 Da). In addition, the other parameters of the mass spectra, such as AI_mod,wa_, DBE_wa_, H/C_wa_, O/C_wa_ CHO, CHON and CHOS, were basically comparable with those of existing studies (Table 1). The main DOM compounds, including the CHO, CHON and CHOS species, were also identified by employing Orbitrap MS [46,47]. Noteworthily, 44 additional CHONS compounds were detected in this study. Molecular formulas including C, H and O are often also taken into account when characterizing SRFA using an MS analysis because of the minor contributions of N and S [11]. In fact, only a few N/S-containing compounds (CHONS) have been detected in the SRFA standard and aquatic DOMs [49,50].

Supplementary Figures S1 and S2 demonstrate that the spectra of the retentate and dialysate were obviously different from those of the SRFA solution after one week of dialysis, a result that is consistent with the DOC and UV results (Figure 1 and Figure 2). As the dialysis progressed, the number and intensity of detected MS peaks in the SRFA retentate gradually decreased, trends that were opposite to those of the dialysate (Supplementary Figures S1 and S2). Most of the MS peaks of SRFA were normally distributed at *m*/*z* 100–600 Da.

**Figure 6:**
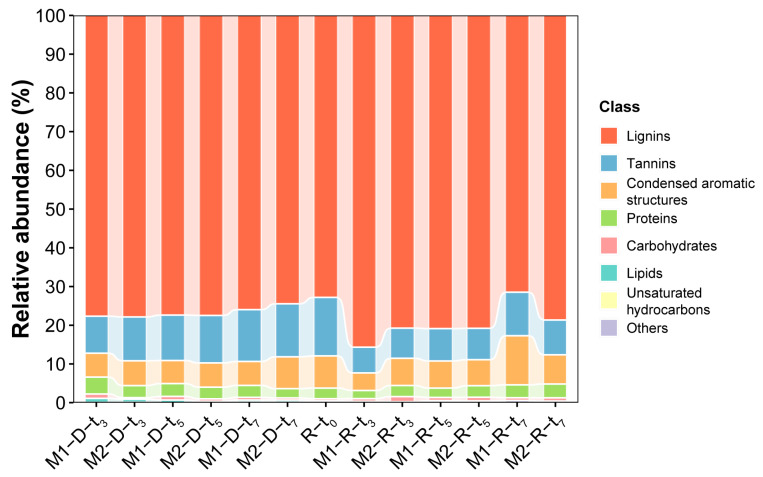
Bar diagrams showing the distribution of classified DOM compounds in the different SRFA solutions. The abbreviations M1 and M2 stand for the 100–500 Da and 500–1000 Da MWCO membranes, respectively; D and R denote the SRFA dialysate and retentate, respectively; and *t*_0_, *t*_3_, *t*_5_ and *t*_7_ represent the samples from the zeroth, third, fifth and seventh days of the dialysis experiment, respectively.

Furthermore, the VK diagrams show the changes in identified DOMs in the retentate and dialysate with increasing dialysis time compared with the original SRFA solution (Supplementary Figures S3–S5). Relatively high-molecular-weight DOM fractions were concentrated in the retentate of the 500–1000 Da MWCO membranes, while more LMW DOM compounds were identified below 500 Da in the dialysate of the 100–500 Da MWCO membrane after one week of dialysis (Figure 3). Specifically, the abundance of most lignins and tannins decreased, while a small portion of them were removed through membrane dialysis, accompanied by an increase in other lignins and condensed aromatic molecules with low O/C and high H/C values (Figure 4 and Supplementary Figure S6). The normalized distribution of different types of DOM compounds during the dialysis is illustrated in Figure 6. The proportion of tannins and condensed aromatic compounds in the retentates first decreased and then increased, while their proportions in the dialysates kept increasing. Compared with the original SRFA solution, the dialysis process definitely led to an increasing abundance of characteristic aromatic/lignin compounds in the final retentate.

**Figure 7:**
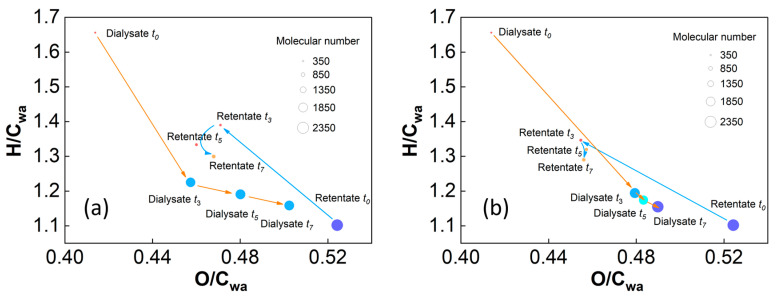
DOM distributions of the SRFA retentate and dialysate using the (**a**) 100–500 Da MWCO membrane and (**b**) 500–1000 Da MWCO membrane. The bubble size indicates the number of DOM molecules; and *t*_0_, *t*_3_, *t*_5_ and *t*_7_ stand for the samples from the zeroth, third, fifth and seventh days of the dialysis experiment.

The trends of the O/C and H/C ratios in the SRFA dialysate of both kinds of cut-off membranes indicated that more DOM molecules with high O/C and low H/C were dialyzed out during the whole experiment (Figure 7a,b). Moreover, the dialysis process occurred mainly in the first three days, with a higher dialysance rate of DOC mass and larger variations in UV–Vis adsorption for the 500–1000 Da MWCO membrane (Figure 1 and Figure 2). Overall, more aromatic rings and phenolic hydroxyl groups with higher O/C_wa_ and lower H/C_wa_ were ultimately retained in the retention solution, and the rest were transferred to the dialysate. The final dialysate contained fewer aromatic compounds and more saturated characteristics than the retentate, which is in accordance with the UV−Vis absorbance results described above (Figure 2 and Supplementary Table S1).

### 3.3. Characterization of LMW DOMs by Combining Membrane Dialysis and Orbitrap MS Analysis

Fulvic acid made up the acid-soluble fraction of the DOMs and accounted for most of the aquatic DOMs, with a molecular weight distribution of 200–2000 Da [11]. More than half of the mass of SRFA passed through both cut-off membranes in the present study, according to our DOC mass calculation. This result means that SRFA was mostly distributed in the LMW fractions (<500 Da). Relatively low-molecular-weight fractions predominated in fulvic acid, while more aliphatic components were seen in the dialysate and more aromatics in the retentate, results that have important implications for the heterogeneous bioavailability and reactivity of DOMs in various aquatic environments. Moreover, the retained and dialysate solutions have markedly striking physicochemical properties, which are supported by their spectral characteristics (Figure 2 and Supplementary Table S1). In fact, the LMW components contribute a substantial proportion to the aquatic DOMs [16]. Unveiling the chemical composition of LMW DOMs is thus essential in order to accurately assess their environmental behavior in natural water bodies.

We further analyzed the MS spectra below the 200 *m*/*z* range because of the high resolution and accuracy of Orbitrap MS analyses in the lower mass region [47]. This conclusion was corroborated by the results of this study: more LMW DOMs, particularly at 70–200 Da, were identified in the dialysate of the 100–500 Da MWCO membrane compared with that in the retentate (Supplementary Figure S2 and Figure 5). The SRFA dialysate was enriched with more aliphatic compounds, such as lipids, peptides and carbohydrates, in the LMW fractions. The carbohydrates and proteins separated from the SRFA were mainly monosaccharides, disaccharides, amino acids and polypeptides. Proteins are compounds formed from a continuous chain of amino acids, joined together by peptide bonds. As protein compounds, peptides usually possess lower molecular weights. More interestingly, 89 and 94 LMW DOMs were detected in the dialysate of the two kinds of membranes on the seventh day, an increase of 59–84% compared with those in the original SRFA. In other words, a new LMW DOM formed throughout the dialysis duration. This could be easily explained by the fact that SRFA comprises LMW components that are present individually or in loosely associated assemblies [11,51]. These small-molecule compounds gradually split during the dialysis. Another possibility was that a small portion of the unstable DOM compounds were degraded after one week of dialysis, alongside a slight carbon loss [35]. Combined with the VK plot partitioning (Figure 5), it was concluded that some lignins, tannins and condensed aromatic structures could be decomposed into small-molecule constituents during the dialysis process. Although membrane dialysis is a simple and efficient method of characterizing LMW DOMs, it still has some drawbacks. Currently, the most commonly used dialysis treatment employs dialysis tubing that is clamped on both ends. This method is susceptible to confounding factors linked to the diffusion of DOMs through the membrane or the interaction of DOMs with the membrane itself. As mentioned earlier, different types of dialysis membranes, dialysis media, substrate concentrations and pH conditions can affect the separation of DOMs to varying degrees [34,35,36]. In a future study, the dialysis rate needs to be further improved by reducing the small amount of DOM compounds adsorbed on the membrane. Particularly, more high-quality dialysis membranes should be developed for selectively separating specific molecular weight fragments of DOMs.

It is worth mentioning that numerous FT-ICR MS studies on DOM composition have not considered LMW fractions below 200 Da [23,26,35]. However, one advantage of Orbitrap MS happens to be the ability to assess ionic fragments at the low *m*/*z* range [11]; Orbitrap MS has also been proven to be capable of characterizing the LMW components of SRFA and various aquatic DOMs with low heteroatom (N, S and P) contents [11]. The Bray–Curtis dissimilarity (2.85 ± 0.42%) of Orbitrap MS between duplicate measurements has demonstrated an excellent reproducibility for DOM characterization [25]. However, the potential limitations of Orbitrap MS also need to be pointed out. Firstly, its lower resolution than FT-ICR MS must be noted. However, while FT-ICR has a better resolution, Orbitrap MS can be an affordable alternative for the preliminary characterization of aquatic DOM samples [25,46,47]. Secondly, Orbitrap MS may only be more suitable than FT-ICR MS when characterizing DOMs containing fewer heteroatoms (N, S and P) [11,26].

LMW DOMs constitute thousands of different compounds and contribute a substantial proportion to aquatic DOMs [11,23]. Moreover, LMW DOMs generally exert distinctive aromaticity, hydrophobicity and bioactivity compared with HMW DOMs and, thus, play vital roles in regulating the transportation, transformation and fate of organic or inorganic contaminants in aquatic environments [14,15,16]. In this study, carbohydrates, lipids and peptides dominated the LMW DOM component in the SRFA dialysate, and more aliphatic characteristics manifested compared with in the initial SRFA solution. This conclusion was further reinforced by the UV–Vis adsorption spectroscopy and high-resolution MS analysis results, as discussed above. The high reactivity of LMW DOMs was further demonstrated by the present study. Future studies need to focus on the important role of LMW DOMs in the biogeochemical processes of contaminants in aquatic ecosystems. In short, this study developed a practical dialysis pretreatment, in combination with Orbitrap MS analysis, for LMW DOM quantification in aquatic environments.

## 4. Materials and Methods

### 4.1. Samples and Reagents

Through the IHSS, we obtained the SRFA (2S101F) standard [40]. Suwannee River, originating in the Okefenokee peat swamp, contains large amounts of DOM molecules. By collecting the blackwater from this river, SRFA was developed as a reference for aquatic humus substances, as it facilitates DOM-related research in aquatic environments. Owing to its low heteroatom content, SRFA can be well selected for Orbitrap MS. Each solution was prepared using ultrapure water (18.0 MΩ·cm).

### 4.2. Membrane Dialysis

For the SRFA, a dialysis experiment was carried out using two kinds of cellulose ester membranes (American Spectrum Medicine Boston, MA, USA) with MWCOs of 100–500 Da and 500–1000 Da. The ends of the membrane were clamped using special dialysis tubing clips (60 mm), and a 4 mL SRFA solution was added to the tubes. The dialysis tube was soaked in a beaker filled with 80 mL of ultrapure water, and the bottom of the beaker was stirred using a magnet. The beaker was wrapped completely in aluminum foil to avoid sunlight radiation. On the third day, 100 μL of the solution inside the membrane was removed and labeled the retentate solution, 10 mL of the same solution but outside the membrane was obtained and labeled as the dialysate, and 10 mL of fresh ultrapure water was added outside the membrane. These sampling steps were repeated on the fifth and seventh days.

### 4.3. Determination of Dissolved Organic Carbon (DOC)

For the initial, retained and dialyzed solutions, we quantified their DOC concentrations with a total organic carbon analyzer (Aurora 1030W, OI Analytical, College Station, TX, USA). All samples to be tested were kept in brown glass bottles at volumes of more than 30 mL. Each sample was determined at least thrice in parallel.

### 4.4. Ultraviolet−Visible Absorption Spectra

The UV–Vis absorption spectra were obtained with a spectrophotometer (BlueStar A, LabTech, Sorisole, Italy). In order to ensure appropriate absorbance values for the DOMs in the SRFA retentate and dialysate, the experimental solutions from the membrane dialysis were diluted to similar DOC concentrations before UV–Vis characterization. The dialysates (*t*_3_, *t*_5_ and *t*_7_) were diluted twice, while the retentates (*t*_0_, *t*_3_, *t*_5_ and *t*_7_) taken at different time intervals were diluted 160–300 times. We selected ultrapure water as the blank control to remove Raman scattering. After the quartz cuvette was moistened with the sample, the sample solutions were slowly added to avoid bubbles. The scanning range was 200 to 800 nm, with a 1 nm scanning interval. The absorbances were calculated back to those of the undiluted samples [52,53]. The absorption coefficients of the chromophoric DOMs were determined at 254 nm and 280 nm [54]. The absorbance per unit of organic carbon content (SUVA) was also calculated at certain wavelengths [55]. The absorption coefficient ratios at 250-to-365 nm (*E2/E3*), 300-to-400 nm (*E3/E4*) and 465-to-665 nm (*E4/E6*) were calculated according to the methods in previous studies [56,57].

### 4.5. Mass Spectrometry Characterization

The SRFA retentate and dialysate were analyzed via Orbitrap MS (Q Exactive, Thermo Scientific, Waltham, MA, USA) equipped with ultra-high performance liquid chromatography (U3000, Thermo Scientific, Waltham, MA, USA). For external calibration, Orbitrap MS was performed with the manufacturer-specified calibration mixture. Negative-ion mode was used with an electron spray ionization (ESI) source at a mass range of *m*/*z* 70–1000. The operating parameters of the Orbitrap MS instrument were as follows: resolution, 140,000; spray voltage, 2.8 KV; capillary temperature, 350 °C; heater temperature, 300 °C; sheath gas flow rate, 35 psi; auxiliary gas flow rate, 10 arb; s-lens RF level, 50; and AGC target, 1 × 10^6^.

The mass lists of the Orbitrap MS were calibrated with a solvent sample and extracted at 0.2-to-0.4 min, using an Xcalibur Qual Browser (Thermo Scientific, Waltham, MA, USA). The molecular formulas of the DOMs were processed via the software ICBM-OCEAN (V1.0) and assigned by referring to previous studies [58]. The criteria for selecting the chemically possible formulas were as follows: C ≤ 50; H ≤ 100; O ≤ 30; N ≤ 2; S ≤ 2; O/C ≤ 1.2; 0.3 ≤ H/C ≤ 2.2; and −1 < DBE < 50 [26,46]. The VK plots were drawn with the H/C and O/C ratios as the axes, and the plots were distinguished according to their different *m*/*z* ratio intervals. Also, based on the H/C and O/C ratios [59,60], the VK diagram was divided into seven discrete regions: lipids (1.5 ≤ H/C ≤ 2.0, 0 ≤ O/C ≤ 0.3), proteins/peptides (1.5 ≤ H/C ≤ 2.2, 0.3 < O/C ≤ 0.67), carbohydrate (1.5 ≤ H/C ≤ 2.4, 0.67 < O/C ≤ 1.2), unsaturated hydrocarbon (0.7 ≤ H/C < 1.5, 0 ≤ O/C ≤ 0.1), lignins (0.7 ≤ H/C < 1.5, 0.1 < O/C ≤ 0.67), tannins (0.5 ≤ H/C < 1.5, 0.67 < O/C ≤ 1.2) and condensed aromatic molecules (0.2 ≤ H/C < 0.7, 0 ≤ O/C ≤ 0.67). The normalized intensities were calculated by dividing the individual peak by the total intensities of all assigned molecular formulas in the MS spectra.

## 5. Conclusions

This study combined a membrane dialysis, UV–Vis absorption spectra and high-resolution Orbitrap MS to separate and identify LMW DOM constituents using a reference standard of SRFA. After one week of dialysis, a significant fraction of SRFA eventually passed through two kinds of cut-off membranes. Moreover, the dialysis rate of the 500–1000 Da MWCO membrane was noticeably higher than that of the 100–500 Da MWCO membrane, indicating the predominance of small molecular components in SRFA. The compounds in the retentate contained more aromatic rings and phenolic hydroxyls with higher O/C_wa_ and lower H/C_wa_, while more aliphatic chains with higher H/C_wa_ and lower O/C_wa_ were enriched in the dialysate. These differences were further reinforced by the UV−Vis absorbance results. It is important to point out that LMW DOM components (<200 Da) were successfully identified in the dialysate via Orbitrap MS due to their low *m*/*z* scan range, high resolution and good precision. Thus, this study demonstrated that optional membrane dialysis in conjunction with high-resolution Orbitrap MS can be practically applied in the characterization of LMW DOMs in aquatic environments.

## Figures and Tables

**Table 1:** Comparison of the molecular composition of SRFA obtained with Orbitrap MS in ESI negative-ion mode *.

*c* (mg·L^−1^)	*n*	*m*/*z*_wa_	AI_mod,wa_	DBE_wa_	H /C_wa_	O /C_wa_	CHO	CHON	CHONS	CHOS	Instrument Parameters			Ref.
											Model	R (e4)	*m*/*z*	
20	3675	472.28	0.40	12.15	1.00	0.49	3141	495	0	39	OE	14	150–1000	[46]
20	3412	457.91	0.40	11.65	1.00	0.51	2696	539	0	177	OV	14		
20	5036	455.78	0.41	11.99	0.97	0.50	3490	1114	0	432	OV	48		
20	2280	407.92	0.42	10.84	1.00	0.45	1822	347	0	111	QE	24		
20	3487	454.77	0.39	11.46	1.01	0.50	2602	663	0	222	OV	24		
20	1870	437.64	0.41	11.47	0.97	0.53	1705	127	0	38	QE	14		
20	3301	495.45	0.41	12.97	0.97	0.50	2913	339	0	49	OE	NG		
50	4350–7029	368.69–454.48	0.36–0.42	9.58–12.01	0.92–1.04	0.57–0.65	NG	NG	NG	NG	OF	50	150–800	[47]
67	3935	355.19	0.35	8.60	1.09	0.53	2689	913	44	289	QE	14	70–1050	This study

* Solution concentrations (*c*). Number of identified molecules (*n*). Not given (NG). Resolution ratio (R). Weight-averaged values were used for mass-to-charge ratio (*m*/*z*_wa_), modified aromatic index (AI_mod,wa_), double-bond equivalent (DBE_wa_), hydrogen-to-carbon ratio (H/C_wa_), and oxygen-to-carbon ratio (O/C_wa_). The CHO, CHON, CHONS and CHOS columns represent the number of compounds containing those elements. The abbreviations for the mass spectrometer models are as follows: Orbitrap Elite (OE), Orbitrap Velos (OV), Orbitrap Q Exactive (QE) and Orbitrap Fusion (OF).

## Data Availability

Data are contained within the article and Supplementary Materials.

## Supplementary Materials

The following supporting information can be downloaded at https://www.mdpi.com/article/10.3390/molecules29143370/s1, Figure S1: Full MS spectra of the SRFA retentate from the 100–500 Da MWCO membrane on the (a) 3rd, (b) 5th and (c) 7th days; Figure S2: Full MS spectra of the SRFA dialysate from different membranes on the 5th day; Figure S3: VK plots of identified DOM with *m*/*z* 70–800 in the original SRFA solution, with colors indicating normalized peak intensities; Figure S4: VK plots for identified DOM with *m*/*z* 70–800 in the SRFA retentate; Figure S5: VK plots for identified DOM with *m*/*z* 70–800 in the SRFA dialysate; Figure S6: VK plots for DOM compounds with *m*/*z* 70–800 in the SRFA solution using 500–1000 Da MWCO membrane. Table S1: The optical parameters of UV-Vis spectra of the SRFA retentate and dialysate; Table S2: Content comparison of the dissolved organic matter after dialysis; Table S3: Content, magnitude and types comparison of the DOM after dialysis; Table S4: The formulas of identified LMW-DOM in the initial SRFA solution (Figure 5a); Table S5: The formulas of identified LMW-DOM in the initial SRFA solution (Figure 5b); Table S6: The formulas of identified LMW-DOM in the retentate from the 100–500 Da MWCO membrane on the 7th day (Figure 5c); Table S7: The formulas of identified LMW-DOM in the retentate from the 500–1000 Da MWCO membrane on the 7th day (Figure 5d); Table S8: The formulas of identified LMW-DOM in the dialysate from the 100–500 Da MWCO membrane on the 7th day (Figure 5e); Table S9: The formulas of identified LMW-DOM in the dialysate from the 500–1000 Da MWCO membrane on the 7th day (Figure 5f).
