# Supplementary material for: Characterization of Low-Molecular-Weight Dissolved Organic Matter Using Optional Dialysis and Orbitrap Mass Spectrometry
Source: Molecules. 2024 Jul 18;29(14):3370. doi: 10.3390/molecules29143370 (PMC11280429; doi:10.3390/molecules29143370)
Supplement: Supplementary file 1 [file molecules-29-03370-s001.zip › molecules-3050138-supplementary.pdf]

Supplementary Materials

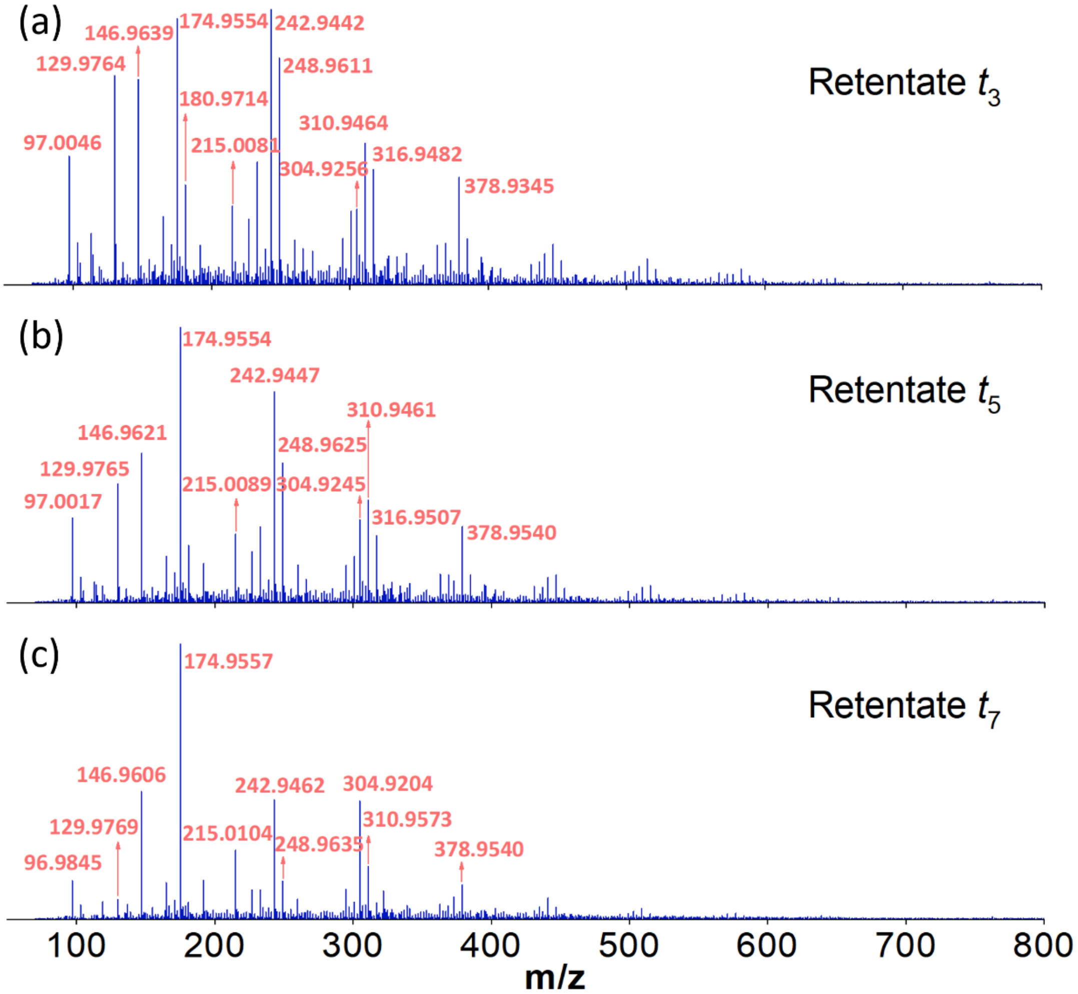

Figure S1. Full MS spectra of the SRFA retentate from 100-500 Da MWCO membrane on the (a) 3rd, (b) 5th and (c) 7th days.

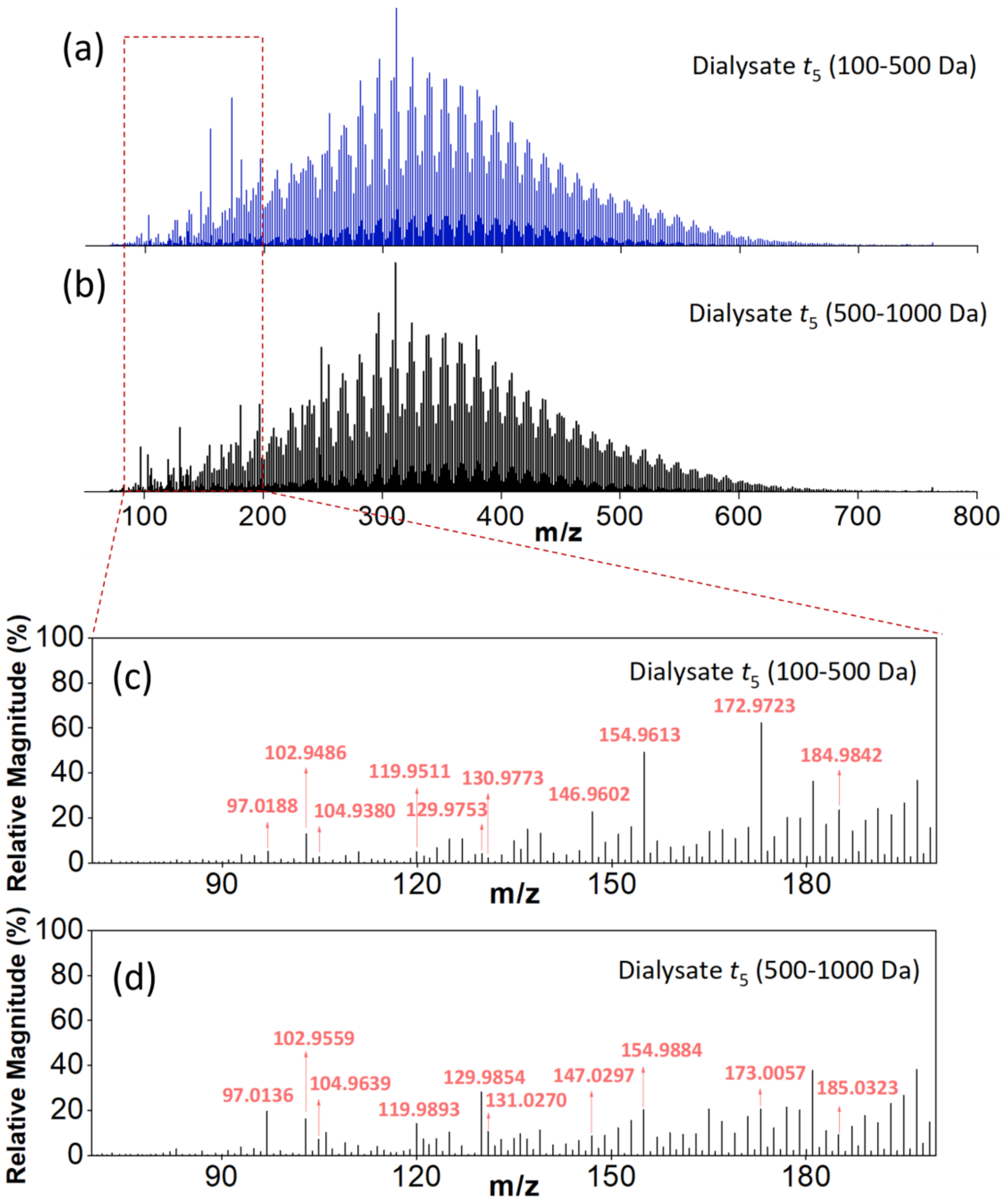

Figure S2. Full MS spectra of SRFA dialysate from (a) 100-500 Da and (b) 500-1000 Da MWCO membranes on the 5th day; (c) and (d) are regional features at m/z 70-200 Da of (a) and (b), respectively.

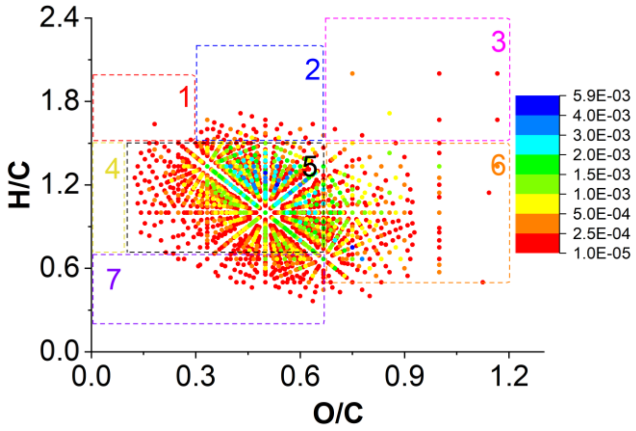

Figure S3. VK plots of identified DOMs with m/z 70-800 in the original SRFA solution, with colors indicating normalized peak intensities. The numbers in the figure represent (1) lipids, (2) proteins (including peptides), (3) carbohydrates, (4) unsaturated hydrocarbons, (5) lignins, (6) tannins, and (7) condensed aromatic molecules.

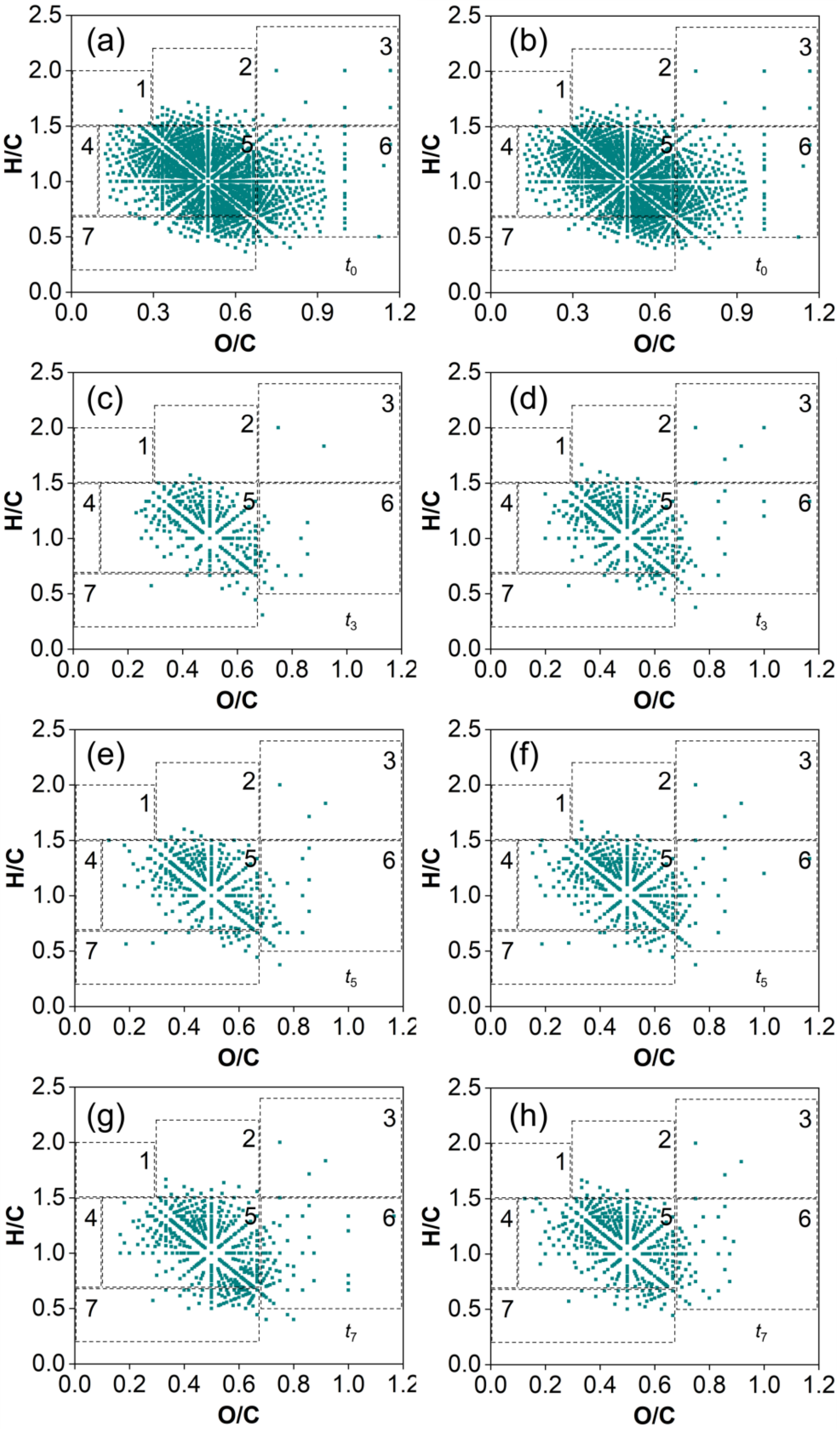

Figure S4. VK plots for identified DOMs with m/z 70-800 in the SRFA retentate. (a, b) Initial SRFA solution, (c, e, g) the retentate from 100-500 Da MWCO and (d, f, h) from 500-1000 Da MWCO membranes at different intervals same as previously labeled. Compound classification marks (1-7) as above.

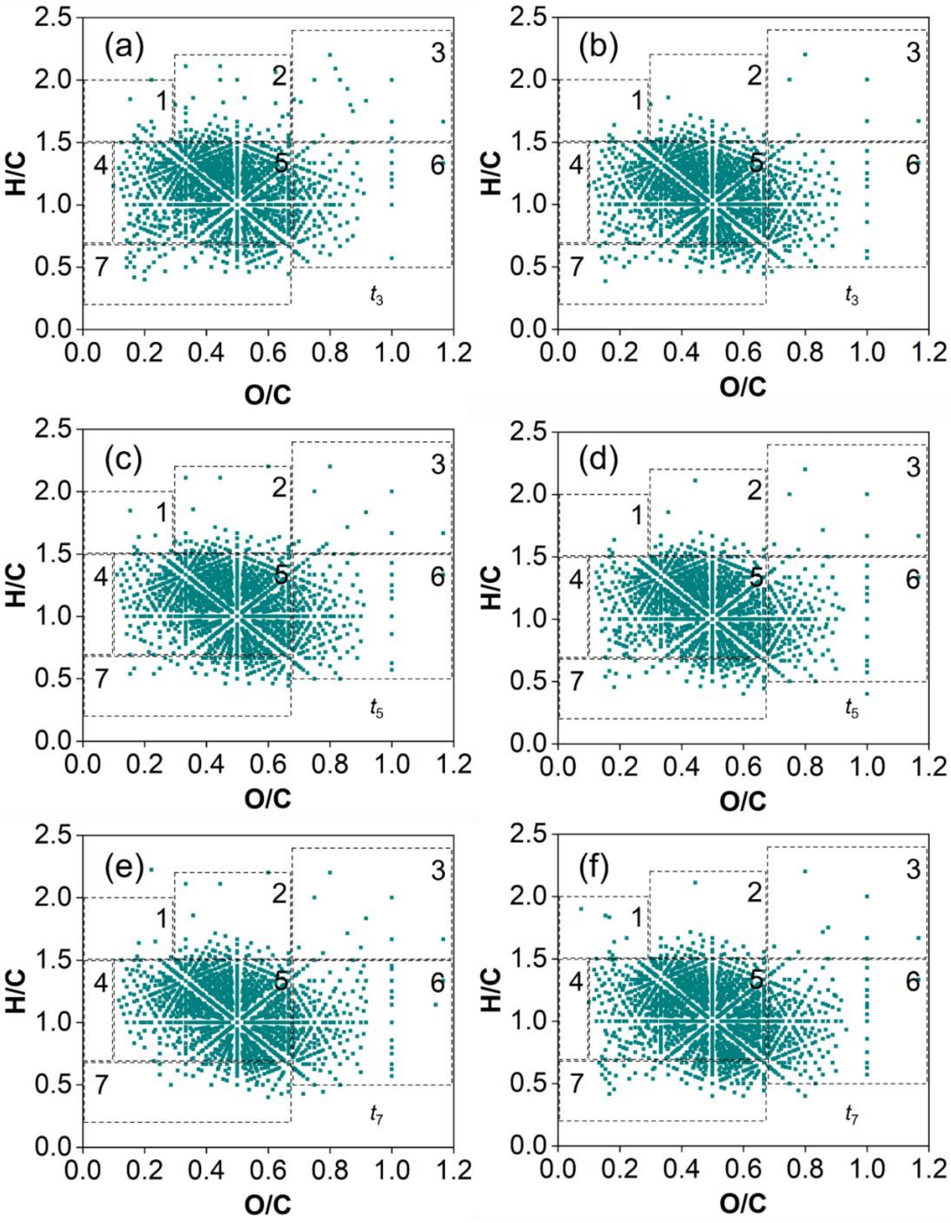

Figure S5. VK plots for identified DOMs with m/z 70-800 in the SRFA dialysate. (a, c, e) The dialysate from 100-500 Da MWCO membrane and (b, d, f) from 500-1000 Da MWCO membrane at different intervals. Compound classification marks (1-7) as above.

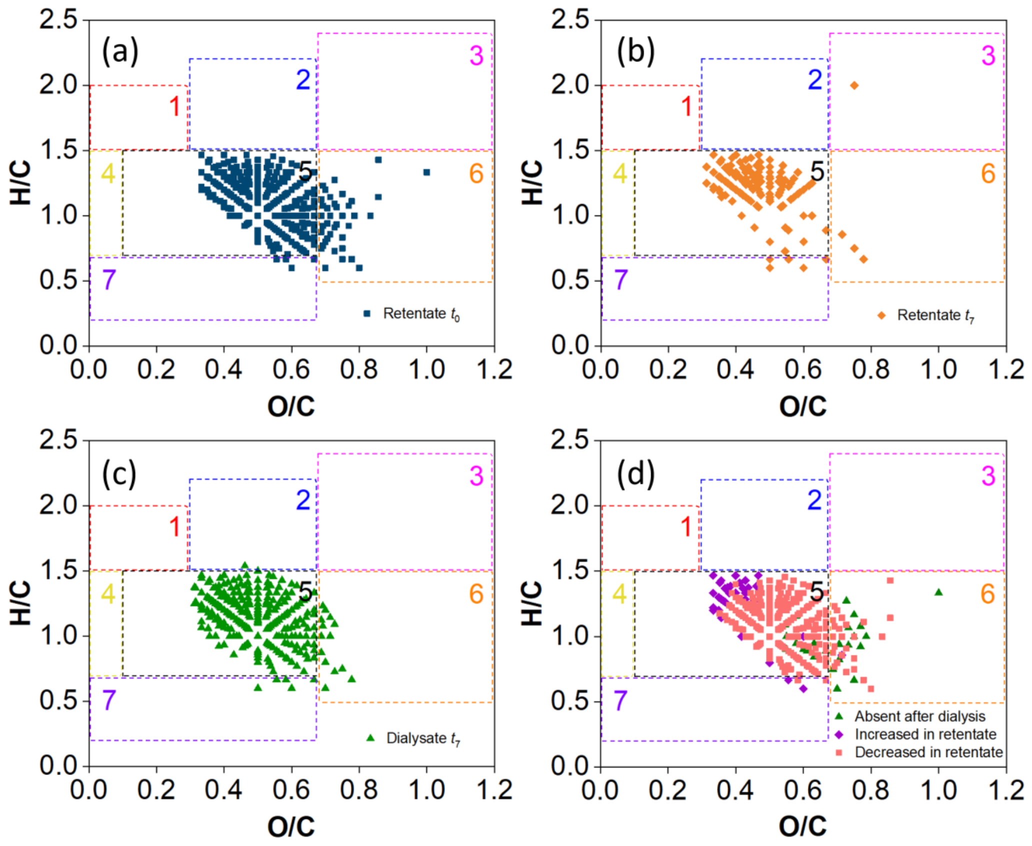

Figure S6. VK plots for DOM compounds with m/z 70-800 in the SRFA solution using 500-1000 Da MWCO membrane. (a) Initial SRFA solution; (b, c) the retentate and dialysate on the 7th day; (d) changes of compounds in the SRFA solution after dialysis. Only compounds with normalised magnitudes higher than 0.001 in the mass spectra were shown, representing between 12% and 26% of the corresponding identified peaks. Compound classification marks (1-7) as above.

Table S1. The optical parameters of UV-Vis spectra of the SRFA retentate and dialysate.

Sample		a_{254}/m^{-1}	a_{280}/m^{-1}	SUVA$_{254}$/ (L·mg^{-1}·m^{-1})	SUVA$_{280}$/ (L·mg^{-1}·m^{-1})	$E2/E3$	$E3/E4$	$E4/E6$
100-500 Da MWCO membrane	Retentate t_0	93.64	70.76	9.07	6.86	3.61	3.68	2.00
	Retentate t_3	31.24	25.41	7.67	6.24	2.67	2.56	1.61
	Retentate t_5	27.48	22.41	8.06	6.57	2.62	2.46	1.45
	Retentate t_7	26.37	21.32	9.04	7.31	2.78	2.70	1.66
	Dialysate t_0	0.15	0.08	2.02	1.12	3.64	1.19	2.03
	Dialysate t_3	33.48	22.98	6.77	4.65	5.45	6.14	2.76
	Dialysate t_5	38.33	26.72	7.38	5.15	5.15	5.70	2.52
	Dialysate t_7	41.47	29.11	7.61	5.34	5.04	5.59	2.43
500-1000 Da MWCO membrane	Retentate t_0	93.64	70.76	9.07	6.86	3.61	3.68	2.00
	Retentate t_3	31.22	25.47	7.78	6.34	2.56	2.43	1.53
	Retentate t_5	27.50	22.52	8.41	6.89	2.50	2.36	1.56
	Retentate t_7	22.61	18.52	8.68	7.11	2.60	2.50	1.63
	Dialysate t_0	0.15	0.08	2.02	1.12	3.64	1.19	2.03
	Dialysate t_3	45.01	31.66	7.90	5.56	4.97	5.63	3.43
	Dialysate t_5	53.51	38.25	8.67	6.20	4.70	5.32	3.36
	Dialysate t_7	57.15	41.07	8.79	6.32	4.59	5.16	3.02

Table S2. Content comparison of the dissolved organic matter after dialysis.

Sample		Quartile of molecular weight (Da)				
		0 %	25 %	50 %	75 %	100 %
Original SRFA	Retentate t_0	70.0291	249.0263	385.6734	523.0906	1049.2092
100-500 Da MWCO membrane	Retentate t_7	70.0803	253.0019	428.0012	612.1132	1049.7588
	Dialysate t_7	70.0261	218.9856	352.9878	490.0630	1049.1802
500-1000 Da MWCO membrane	Retentate t_7	70.0377	249.0400	437.0375	647.0286	1049.7633
	Dialysate t_7	70.0193	227.9396	360.0570	499.0097	1049.5722

Table S3. Content, magnitude and types comparison of the DOM after dialysis.

Membrane	Sample	Total number					DOM with normalized magnitude >0.001	Proportion
		DOM	CHO	CHON	CHONS	CHOS		
100-500 Da MWCO	Retentate t_0	2094	1822	259	1	12	281	13.4 %
	Retentate t_7	543	536	4	0	3	122	22.5 %
	Dialysate t_7	1746	1518	202	9	17	257	14.7 %
	Absent after dialysis	1562	1293	257	1	11	19	1.2 %
	Increased in retentate	138	135	2	0	1	37	26.8 %
	Decreased in retentate	1956	1687	257	1	11	227	11.6 %
500-1000 Da MWCO	Retentate t_0	1975	1763	202	0	10	281	14.2 %
	Retentate t_7	477	472	4	0	1	122	25.6 %
	Dialysate t_7	2043	1619	330	34	60	262	12.8 %
	Absent after dialysis	1505	1295	201	0	9	24	1.6 %
	Increased in retentate	104	99	4	0	1	36	34.6 %
	Decreased in retentate	1878	1668	201	0	9	220	11.7 %

Table S4. The formulas of identified LMW-DOM in the initial SRFA solution (Figure 5a).

m/z	group	formula	H/C	O/C	S/C	N/C
125.0477	CHON	$C_6H_7N_1O_2$	1.17	0.33	0	0.17
127.9932	CHOS	$C_5H_4O_2S_1$	0.80	0.40	0.20	0
134.0368	CHO	$C_8H_6O_2$	0.75	0.25	0	0
142.0089	CHOS	$C_6H_6O_2S_1$	1.00	0.33	0.17	0
146.0215	CHO	$C_5H_6O_5$	1.20	1.00	0	0
151.0633	CHON	$C_8H_9N_1O_2$	1.13	0.25	0	0.13
152.0110	CHO	$C_7H_4O_4$	0.57	0.57	0	0
156.0059	CHO	$C_6H_4O_5$	0.67	0.83	0	0
158.0215	CHO	$C_6H_6O_5$	1.00	0.83	0	0
160.0372	CHO	$C_6H_8O_5$	1.33	0.83	0	0
160.0524	CHO	$C_{10}H_8O_2$	0.80	0.20	0	0
162.0681	CHO	$C_{10}H_{10}O_2$	1.00	0.20	0	0
165.0790	CHON	$C_9H_{11}N_1O_2$	1.22	0.22	0	0.11
166.0994	CHO	$C_{10}H_{14}O_2$	1.40	0.20	0	0
168.0059	CHO	$C_7H_4O_5$	0.57	0.71	0	0
170.0038	CHOS	$C_7H_6O_3S_1$	0.86	0.43	0.14	0
170.0215	CHO	$C_7H_6O_5$	0.86	0.71	0	0
171.9830	CHOS	$C_6H_4O_4S_1$	0.67	0.67	0.17	0
172.0372	CHO	$C_7H_8O_5$	1.14	0.71	0	0
174.0164	CHO	$C_6H_6O_6$	1.00	1.00	0	0
174.0681	CHO	$C_{11}H_{10}O_2$	0.91	0.18	0	0
176.0321	CHO	$C_6H_8O_6$	1.33	1.00	0	0
176.0837	CHO	$C_{11}H_{12}O_2$	1.09	0.18	0	0
177.0426	CHON	$C_9H_7N_1O_3$	0.78	0.33	0	0.11
178.0477	CHO	$C_6H_{10}O_6$	1.67	1.00	0	0
180.0059	CHO	$C_8H_4O_5$	0.50	0.63	0	0
180.0634	CHO	$C_6H_{12}O_6$	2.00	1.00	0	0
180.1150	CHO	$C_{11}H_{16}O_2$	1.45	0.18	0	0
182.1307	CHO	$C_{11}H_{18}O_2$	1.64	0.18	0	0
184.0008	CHO	$C_7H_4O_6$	0.57	0.86	0	0
184.0372	CHO	$C_8H_8O_5$	1.00	0.63	0	0
185.0688	CHON	$C_8H_{11}N_1O_4$	1.38	0.50	0	0.13
185.9987	CHOS	$C_7H_6O_4S_1$	0.86	0.57	0.14	0
186.0164	CHO	$C_7H_6O_6$	0.86	0.86	0	0
186.0528	CHO	$C_8H_{10}O_5$	1.25	0.63	0	0
186.0681	CHO	$C_{12}H_{10}O_2$	0.83	0.17	0	0
188.0321	CHO	$C_7H_8O_6$	1.14	0.86	0	0
188.0837	CHO	$C_{12}H_{12}O_2$	1.00	0.17	0	0
190.0477	CHO	$C_7H_{10}O_6$	1.43	0.86	0	0
190.0994	CHO	$C_{12}H_{14}O_2$	1.17	0.17	0	0
192.0270	CHO	$C_6H_8O_7$	1.33	1.17	0	0
192.0634	CHO	$C_7H_{12}O_6$	1.71	0.86	0	0
193.0375	CHON	$C_9H_7N_1O_4$	0.78	0.44	0	0.11
193.0739	CHON	$C_{10}H_{11}N_1O_3$	1.10	0.30	0	0.10
194.0215	CHO	$C_9H_6O_5$	0.67	0.56	0	0
194.0427	CHO	$C_6H_{10}O_7$	1.67	1.17	0	0
194.1307	CHO	$C_{12}H_{18}O_2$	1.50	0.17	0	0
195.0532	CHON	$C_9H_9N_1O_4$	1.00	0.44	0	0.11
196.0008	CHO	$C_8H_4O_6$	0.50	0.75	0	0
196.0372	CHO	$C_9H_8O_5$	0.89	0.56	0	0
196.0583	CHO	$C_6H_{12}O_7$	2.00	1.17	0	0
197.0324	CHON	$C_8H_7N_1O_5$	0.88	0.63	0	0.13
198.0164	CHO	$C_8H_6O_6$	0.75	0.75	0	0

198.0528	CHO	$C_9H_{10}O_5$	1.11	0.56	0	0
199.0117	CHON	$C_7H_5N_1O_6$	0.71	0.86	0	0.14
199.9957	CHO	$C_7H_4O_7$	0.57	1.00	0	0

Table S5. The formulas of identified LMW-DOM in the initial SRFA solution (Figure 5b).

m/z	group	formula	H/C	O/C	S/C	N/C
125.0477	CHON	$C_6H_7N_1O_2$	1.17	0.33	0	0.17
127.9932	CHOS	$C_5H_4O_2S_1$	0.80	0.40	0.20	0
134.0368	CHO	$C_8H_6O_2$	0.75	0.25	0	0
136.0160	CHO	$C_7H_4O_3$	0.57	0.43	0	0
144.0059	CHO	$C_5H_4O_5$	0.80	1.00	0	0
146.0215	CHO	$C_5H_6O_5$	1.20	1.00	0	0
156.0059	CHO	$C_6H_4O_5$	0.67	0.83	0	0
158.0215	CHO	$C_6H_6O_5$	1.00	0.83	0	0
160.0372	CHO	$C_6H_8O_5$	1.33	0.83	0	0
160.0524	CHO	$C_{10}H_8O_2$	0.80	0.20	0	0
162.0681	CHO	$C_{10}H_{10}O_2$	1.00	0.20	0	0
166.0994	CHO	$C_{10}H_{14}O_2$	1.40	0.20	0	0
170.0038	CHOS	$C_7H_6O_3S_1$	0.86	0.43	0.14	0
170.0215	CHO	$C_7H_6O_5$	0.86	0.71	0	0
171.9830	CHOS	$C_6H_4O_4S_1$	0.67	0.67	0.17	0
172.0372	CHO	$C_7H_8O_5$	1.14	0.71	0	0
174.0164	CHO	$C_6H_6O_6$	1.00	1.00	0	0
174.0681	CHO	$C_{11}H_{10}O_2$	0.91	0.18	0	0
176.0321	CHO	$C_6H_8O_6$	1.33	1.00	0	0
176.0837	CHO	$C_{11}H_{12}O_2$	1.09	0.18	0	0
178.0477	CHO	$C_6H_{10}O_6$	1.67	1.00	0	0
179.0219	CHON	$C_8H_5N_1O_4$	0.63	0.50	0	0.13
180.0059	CHO	$C_8H_4O_5$	0.50	0.63	0	0
180.0634	CHO	$C_6H_{12}O_6$	2.00	1.00	0	0
180.1150	CHO	$C_{11}H_{16}O_2$	1.45	0.18	0	0
182.1307	CHO	$C_{11}H_{18}O_2$	1.64	0.18	0	0
184.0372	CHO	$C_8H_8O_5$	1.00	0.63	0	0
185.0324	CHON	$C_7H_7N_1O_5$	1.00	0.71	0	0.14
186.0164	CHO	$C_7H_6O_6$	0.86	0.86	0	0
186.0528	CHO	$C_8H_{10}O_5$	1.25	0.63	0	0
186.0681	CHO	$C_{12}H_{10}O_2$	0.83	0.17	0	0
188.0321	CHO	$C_7H_8O_6$	1.14	0.86	0	0
188.0837	CHO	$C_{12}H_{12}O_2$	1.00	0.17	0	0
190.0477	CHO	$C_7H_{10}O_6$	1.43	0.86	0	0
190.0994	CHO	$C_{12}H_{14}O_2$	1.17	0.17	0	0
191.0582	CHON	$C_{10}H_9N_1O_3$	0.90	0.30	0	0.10
192.0270	CHO	$C_6H_8O_7$	1.33	1.17	0	0
192.0634	CHO	$C_7H_{12}O_6$	1.71	0.86	0	0
193.0375	CHON	$C_9H_7N_1O_4$	0.78	0.44	0	0.11
194.0215	CHO	$C_9H_6O_5$	0.67	0.56	0	0
194.0427	CHO	$C_6H_{10}O_7$	1.67	1.17	0	0
194.1307	CHO	$C_{12}H_{18}O_2$	1.50	0.17	0	0
195.0532	CHON	$C_9H_9N_1O_4$	1.00	0.44	0	0.11
196.0008	CHO	$C_8H_4O_6$	0.50	0.75	0	0
196.0372	CHO	$C_9H_8O_5$	0.89	0.56	0	0
196.0583	CHO	$C_6H_{12}O_7$	2.00	1.17	0	0
197.0324	CHON	$C_8H_7N_1O_5$	0.88	0.63	0	0.13
198.0164	CHO	$C_8H_6O_6$	0.75	0.75	0	0
198.0528	CHO	$C_9H_{10}O_5$	1.11	0.56	0	0
199.0117	CHON	$C_7H_5N_1O_6$	0.71	0.86	0	0.14
199.9957	CHO	$C_7H_4O_7$	0.57	1.00	0	0

Table S6. The formulas of identified LMW-DOM in the retentate from 100-500 Da MWCO membrane on the 7th day (Figure 5c).

m/z	group	formula	H/C	O/C	S/C	N/C
144.0059	CHO	$C_5H_4O_5$	0.80	1.00	0	0
146.0215	CHO	$C_5H_6O_5$	1.20	1.00	0	0
148.0273	CHON	$C_7H_4N_2O_2$	0.57	0.29	0	0.29
152.0110	CHO	$C_7H_4O_4$	0.57	0.57	0	0
156.0059	CHO	$C_6H_4O_5$	0.67	0.83	0	0
158.0215	CHO	$C_6H_6O_5$	1.00	0.83	0	0
160.0372	CHO	$C_6H_8O_5$	1.33	0.83	0	0
162.0681	CHO	$C_{10}H_{10}O_2$	1.00	0.20	0	0
168.0059	CHO	$C_7H_4O_5$	0.57	0.71	0	0
170.0215	CHO	$C_7H_6O_5$	0.86	0.71	0	0
172.0372	CHO	$C_7H_8O_5$	1.14	0.71	0	0
176.0321	CHO	$C_6H_8O_6$	1.33	1.00	0	0
176.0837	CHO	$C_{11}H_{12}O_2$	1.09	0.18	0	0
180.0059	CHO	$C_8H_4O_5$	0.50	0.63	0	0
180.1150	CHO	$C_{11}H_{16}O_2$	1.45	0.18	0	0
184.0372	CHO	$C_8H_8O_5$	1.00	0.63	0	0
186.0528	CHO	$C_8H_{10}O_5$	1.25	0.63	0	0
188.0321	CHO	$C_7H_8O_6$	1.14	0.86	0	0
188.0837	CHO	$C_{12}H_{12}O_2$	1.00	0.17	0	0
190.0477	CHO	$C_7H_{10}O_6$	1.43	0.86	0	0
190.0994	CHO	$C_{12}H_{14}O_2$	1.17	0.17	0	0
192.0270	CHO	$C_6H_8O_7$	1.33	1.17	0	0
192.0634	CHO	$C_7H_{12}O_6$	1.71	0.86	0	0
194.0215	CHO	$C_9H_6O_5$	0.67	0.56	0	0
196.0372	CHO	$C_9H_8O_5$	0.89	0.56	0	0
197.0324	CHON	$C_8H_7N_1O_5$	0.88	0.63	0	0.13
198.0164	CHO	$C_8H_6O_6$	0.75	0.75	0	0
198.0528	CHO	$C_9H_{10}O_5$	1.11	0.56	0	0

Table S7. The formulas of identified LMW-DOM in the retentate from 500-1000 Da MWCO membrane on the 7th day (Figure 5d).

m/z	group	formula	H/C	O/C	S/C	N/C
148.0273	CHON	$C_7H_4N_2O_2$	0.57	0.29	0	0.29
152.0110	CHO	$C_7H_4O_4$	0.57	0.57	0	0
156.0059	CHO	$C_6H_4O_5$	0.67	0.83	0	0
158.0215	CHO	$C_6H_6O_5$	1.00	0.83	0	0
160.0372	CHO	$C_6H_8O_5$	1.33	0.83	0	0
162.0681	CHO	$C_{10}H_{10}O_2$	1.00	0.20	0	0
166.0994	CHO	$C_{10}H_{14}O_2$	1.40	0.20	0	0
168.0059	CHO	$C_7H_4O_5$	0.57	0.71	0	0
170.0215	CHO	$C_7H_6O_5$	0.86	0.71	0	0
172.0372	CHO	$C_7H_8O_5$	1.14	0.71	0	0
174.0681	CHO	$C_{11}H_{10}O_2$	0.91	0.18	0	0
176.0837	CHO	$C_{11}H_{12}O_2$	1.09	0.18	0	0
180.0059	CHO	$C_8H_4O_5$	0.50	0.63	0	0
180.1150	CHO	$C_{11}H_{16}O_2$	1.45	0.18	0	0
184.0372	CHO	$C_8H_8O_5$	1.00	0.63	0	0
186.0528	CHO	$C_8H_{10}O_5$	1.25	0.63	0	0
188.0321	CHO	$C_7H_8O_6$	1.14	0.86	0	0
190.0378	CHON	$C_9H_6N_2O_3$	0.67	0.33	0	0.22
190.0477	CHO	$C_7H_{10}O_6$	1.43	0.86	0	0
192.0634	CHO	$C_7H_{12}O_6$	1.71	0.86	0	0
194.0215	CHO	$C_9H_6O_5$	0.67	0.56	0	0
194.1307	CHO	$C_{12}H_{18}O_2$	1.50	0.17	0	0
196.0372	CHO	$C_9H_8O_5$	0.89	0.56	0	0
198.0164	CHO	$C_8H_6O_6$	0.75	0.75	0	0
198.0528	CHO	$C_9H_{10}O_5$	1.11	0.56	0	0

Table S8. The formulas of identified LMW-DOM in the dialysate from 100-500 Da MWCO membrane on the 7th day (Figure 5e).

m/z	group	formula	H/C	O/C	S/C	N/C
125.0477	CHON	$C_6H_7N_1O_2$	1.17	0.33	0	0.17
127.9932	CHOS	$C_5H_4O_2S_1$	0.80	0.40	0.20	0
134.0368	CHO	$C_8H_6O_2$	0.75	0.25	0	0
136.0160	CHO	$C_7H_4O_3$	0.57	0.43	0	0
144.0059	CHO	$C_5H_4O_5$	0.80	1.00	0	0
146.0215	CHO	$C_5H_6O_5$	1.20	1.00	0	0
149.0477	CHON	$C_8H_7N_1O_2$	0.88	0.25	0	0.13
151.0633	CHON	$C_8H_9N_1O_2$	1.13	0.25	0	0.13
152.0110	CHO	$C_7H_4O_4$	0.57	0.57	0	0
156.0059	CHO	$C_6H_4O_5$	0.67	0.83	0	0
156.0171	CHON	$C_5H_4N_2O_4$	0.80	0.80	0	0.40
158.0215	CHO	$C_6H_6O_5$	1.00	0.83	0	0
160.0372	CHO	$C_6H_8O_5$	1.33	0.83	0	0
160.0524	CHO	$C_{10}H_8O_2$	0.80	0.20	0	0
162.0681	CHO	$C_{10}H_{10}O_2$	1.00	0.20	0	0
166.0994	CHO	$C_{10}H_{14}O_2$	1.40	0.20	0	0
168.0059	CHO	$C_7H_4O_5$	0.57	0.71	0	0
170.0038	CHOS	$C_7H_6O_3S_1$	0.86	0.43	0.14	0
170.0215	CHO	$C_7H_6O_5$	0.86	0.71	0	0
171.9830	CHOS	$C_6H_4O_4S_1$	0.67	0.67	0.17	0
172.0194	CHOS	$C_7H_8O_3S_1$	1.14	0.43	0.14	0
172.0372	CHO	$C_7H_8O_5$	1.14	0.71	0	0
174.0164	CHO	$C_6H_6O_6$	1.00	1.00	0	0
174.0681	CHO	$C_{11}H_{10}O_2$	0.91	0.18	0	0
176.0321	CHO	$C_6H_8O_6$	1.33	1.00	0	0
176.0837	CHO	$C_{11}H_{12}O_2$	1.09	0.18	0	0
177.0426	CHON	$C_9H_7N_1O_3$	0.78	0.33	0	0.11
178.0477	CHO	$C_6H_{10}O_6$	1.67	1.00	0	0
179.0582	CHON	$C_9H_9N_1O_3$	1.00	0.33	0	0.11
180.0059	CHO	$C_8H_4O_5$	0.50	0.63	0	0
180.0634	CHO	$C_6H_{12}O_6$	2.00	1.00	0	0
180.1150	CHO	$C_{11}H_{16}O_2$	1.45	0.18	0	0
181.0409	CHONS	$C_5H_{11}N_1O_4S_1$	2.20	0.80	0.20	0.20
182.1307	CHO	$C_{11}H_{18}O_2$	1.64	0.18	0	0
184.0372	CHO	$C_8H_8O_5$	1.00	0.63	0	0
185.0688	CHON	$C_8H_{11}N_1O_4$	1.38	0.50	0	0.13
186.0164	CHO	$C_7H_6O_6$	0.86	0.86	0	0
186.0528	CHO	$C_8H_{10}O_5$	1.25	0.63	0	0
186.0681	CHO	$C_{12}H_{10}O_2$	0.83	0.17	0	0
188.0321	CHO	$C_7H_8O_6$	1.14	0.86	0	0
188.0837	CHO	$C_{12}H_{12}O_2$	1.00	0.17	0	0
190.0477	CHO	$C_7H_{10}O_6$	1.43	0.86	0	0
190.0994	CHO	$C_{12}H_{14}O_2$	1.17	0.17	0	0
192.0270	CHO	$C_6H_8O_7$	1.33	1.17	0	0
192.0634	CHO	$C_7H_{12}O_6$	1.71	0.86	0	0
193.0375	CHON	$C_9H_7N_1O_4$	0.78	0.44	0	0.11
193.0739	CHON	$C_{10}H_{11}N_1O_3$	1.10	0.30	0	0.10
194.0215	CHO	$C_9H_6O_5$	0.67	0.56	0	0
194.0427	CHO	$C_6H_{10}O_7$	1.67	1.17	0	0
194.1307	CHO	$C_{12}H_{18}O_2$	1.50	0.17	0	0
195.0532	CHON	$C_9H_9N_1O_4$	1.00	0.44	0	0.11

196.0008	CHO	$C_8H_4O_6$	0.50	0.75	0	0
196.0372	CHO	$C_9H_8O_5$	0.89	0.56	0	0
197.0180	CHONS	$C_5H_{11}N_1O_3S_2$	2.20	0.60	0.40	0.20
197.0324	CHON	$C_8H_7N_1O_5$	0.88	0.63	0	0.13
197.0688	CHON	$C_9H_{11}N_1O_4$	1.22	0.44	0	0.11
198.0164	CHO	$C_8H_6O_6$	0.75	0.75	0	0
198.0528	CHO	$C_9H_{10}O_5$	1.11	0.56	0	0
199.0117	CHON	$C_7H_5N_1O_6$	0.71	0.86	0	0.14
199.0481	CHON	$C_8H_9N_1O_5$	1.13	0.63	0	0.13
199.9957	CHO	$C_7H_4O_7$	0.57	1.00	0	0

Table S9. The formulas of identified LMW-DOM in the dialysate from 500-1000 Da MWCO membrane on the 7th day (Figure 5f).

m/z	group	formula	H/C	O/C	S/C	N/C
125.0477	CHON	$C_6H_7N_1O_2$	1.17	0.33	0	0.17
127.9932	CHOS	$C_5H_4O_2S_1$	0.80	0.40	0.20	0
134.0368	CHO	$C_8H_6O_2$	0.75	0.25	0	0
136.0160	CHO	$C_7H_4O_3$	0.57	0.43	0	0
143.9881	CHOS	$C_5H_4O_3S_1$	0.80	0.60	0.20	0
146.0215	CHO	$C_5H_6O_5$	1.20	1.00	0	0
148.0273	CHON	$C_7H_4N_2O_2$	0.57	0.29	0	0.29
149.0477	CHON	$C_8H_7N_1O_2$	0.88	0.25	0	0.13
152.0110	CHO	$C_7H_4O_4$	0.57	0.57	0	0
156.0059	CHO	$C_6H_4O_5$	0.67	0.83	0	0
158.0215	CHO	$C_6H_6O_5$	1.00	0.83	0	0
160.0372	CHO	$C_6H_8O_5$	1.33	0.83	0	0
160.0524	CHO	$C_{10}H_8O_2$	0.80	0.20	0	0
161.0477	CHON	$C_9H_7N_1O_2$	0.78	0.22	0	0.11
162.0681	CHO	$C_{10}H_{10}O_2$	1.00	0.20	0	0
166.0994	CHO	$C_{10}H_{14}O_2$	1.40	0.20	0	0
168.0059	CHO	$C_7H_4O_5$	0.57	0.71	0	0
170.0038	CHOS	$C_7H_6O_3S_1$	0.86	0.43	0.14	0
170.0215	CHO	$C_7H_6O_5$	0.86	0.71	0	0
171.0532	CHON	$C_7H_9N_1O_4$	1.29	0.57	0	0.14
171.9830	CHOS	$C_6H_4O_4S_1$	0.67	0.67	0.17	0
172.0194	CHOS	$C_7H_8O_3S_1$	1.14	0.43	0.14	0
172.0372	CHO	$C_7H_8O_5$	1.14	0.71	0	0
174.0164	CHO	$C_6H_6O_6$	1.00	1.00	0	0
174.0681	CHO	$C_{11}H_{10}O_2$	0.91	0.18	0	0
176.0321	CHO	$C_6H_8O_6$	1.33	1.00	0	0
176.0837	CHO	$C_{11}H_{12}O_2$	1.09	0.18	0	0
177.0426	CHON	$C_9H_7N_1O_3$	0.78	0.33	0	0.11
178.0378	CHON	$C_8H_6N_2O_3$	0.75	0.38	0	0.25
178.0477	CHO	$C_6H_{10}O_6$	1.67	1.00	0	0
179.0219	CHON	$C_8H_5N_1O_4$	0.63	0.50	0	0.13
179.0582	CHON	$C_9H_9N_1O_3$	1.00	0.33	0	0.11
180.0059	CHO	$C_8H_4O_5$	0.50	0.63	0	0
180.0634	CHO	$C_6H_{12}O_6$	2.00	1.00	0	0
180.1150	CHO	$C_{11}H_{16}O_2$	1.45	0.18	0	0
181.0409	CHONS	$C_5H_{11}N_1O_4S_1$	2.20	0.80	0.20	0.20
182.1307	CHO	$C_{11}H_{18}O_2$	1.64	0.18	0	0
184.0008	CHO	$C_7H_4O_6$	0.57	0.86	0	0
184.0372	CHO	$C_8H_8O_5$	1.00	0.63	0	0
185.0324	CHON	$C_7H_7N_1O_5$	1.00	0.71	0	0.14
185.0688	CHON	$C_8H_{11}N_1O_4$	1.38	0.50	0	0.13
186.0164	CHO	$C_7H_6O_6$	0.86	0.86	0	0
186.0528	CHO	$C_8H_{10}O_5$	1.25	0.63	0	0
186.0681	CHO	$C_{12}H_{10}O_2$	0.83	0.17	0	0
188.0321	CHO	$C_7H_8O_6$	1.14	0.86	0	0
188.0837	CHO	$C_{12}H_{12}O_2$	1.00	0.17	0	0
190.0477	CHO	$C_7H_{10}O_6$	1.43	0.86	0	0
190.0994	CHO	$C_{12}H_{14}O_2$	1.17	0.17	0	0
191.0219	CHON	$C_9H_5N_1O_4$	0.56	0.44	0	0.11
192.0270	CHO	$C_6H_8O_7$	1.33	1.17	0	0
192.0634	CHO	$C_7H_{12}O_6$	1.71	0.86	0	0

193.0375	CHON	$C_9H_7N_1O_4$	0.78	0.44	0	0.11
193.0739	CHON	$C_{10}H_{11}N_1O_3$	1.10	0.30	0	0.10
194.0215	CHO	$C_9H_6O_5$	0.67	0.56	0	0
194.0402	CHOS	$C_{10}H_{10}O_2S_1$	1.00	0.20	0.10	0
194.0427	CHO	$C_6H_{10}O_7$	1.67	1.17	0	0
194.1307	CHO	$C_{12}H_{18}O_2$	1.50	0.17	0	0
195.0532	CHON	$C_9H_9N_1O_4$	1.00	0.44	0	0.11
196.0008	CHO	$C_8H_4O_6$	0.50	0.75	0	0
196.0372	CHO	$C_9H_8O_5$	0.89	0.56	0	0
197.0324	CHON	$C_8H_7N_1O_5$	0.88	0.63	0	0.13
197.0688	CHON	$C_9H_{11}N_1O_4$	1.22	0.44	0	0.11
198.0164	CHO	$C_8H_6O_6$	0.75	0.75	0	0
198.0528	CHO	$C_9H_{10}O_5$	1.11	0.56	0	0
198.1620	CHO	$C_{12}H_{22}O_2$	1.83	0.17	0	0
199.0117	CHON	$C_7H_5N_1O_6$	0.71	0.86	0	0.14
199.0481	CHON	$C_8H_9N_1O_5$	1.13	0.63	0	0.13
199.0845	CHON	$C_9H_{13}N_1O_4$	1.44	0.44	0	0.11
199.9957	CHO	$C_7H_4O_7$	0.57	1.00	0	0
